# Quantification of Toxic Chemicals in Selected Human Populations

**DOI:** 10.6028/jres.093.098

**Published:** 1988-06-01

**Authors:** J. S. Holler, D. G. Patterson, S. J. Smith

**Affiliations:** Center for Environmental Health and Injury Control, Centers for Disease Control, Public Health Service, U.S. Department of Health and Human Services, Atlanta, GA 30333

The evaluation of risk posed by toxic chemicals to human populations requires a knowledge of the toxicity of compounds and the extent of human exposure to the compounds. The best estimate of exposure is obtained by measuring chemical residues or metabolites in biological samples and extrapolating the measurements to body burdens in study populations. Analytical methods developed at the Centers for Disease Control (CDC) typify the laboratory techniques needed to support epidemiologic studies. In these procedures, specific sample preparation techniques are used to isolate the target compounds, which are then measured by capillary gas chromatography combined with mass spectrometry. Many problems must be solved for these techniques to be applied successfully. Quality control materials must be generated that closely mimic actual specimens and that contain target analytes at appropriate concentrations. Sample contamination, both from internal and external sources, can be a major difficulty. The lack of analytical standards is usually a continuing problem for the analytical chemist, and a successful synthesis program can minimize this problem. Interpreting analytical results at or near the system’s limit of detection poses more problems. Techniques must be developed to translate sample concentrations into valid estimates of total body-burden values.

A recent study in which chlorinated phenols and phenoxy acid herbicides in urine were measured exemplifies typical laboratory challenges in trace analysis. In this study, we compared children living near a chemical manufacturing site with a control group of unexposed children. An extensive sample preparation included acid hydrolysis, extraction with benzene, derivatization with diazoethane and column chromatography cleanup. The more volatile compounds are quantified by capillary column gas chromatography/positive chemical ionization/mass spectrometry/mass spectrometry. Less volatile compounds are quantified by using electron capture negative chemical ionization in a single stage mass spectrometry mode. This study required a modified approach to quality control material in that target values were obtained during the analysis of actual specimens. In addition, we have used a multivariate quality control procedure to obtain a single quality control chart that is representative of the 12 study analytes. The synthesis of the pure derivatives provided an ideal material in estimating recovery and diagnosing chromatographic problems. With the method’s improved detection limits and specificity, we had an increased frequency of detection for several of the study analytes compared with frequencies for previous studies.

Chemists have encountered significant analytical challenges in measuring 2,3,7,8-tetrachlorodibenzodioxin (TCDD) in tissue at the parts per trillion (ppt) level and measuring TCDD in serum at the parts per quadrillion (ppq) level. Such low levels of quantification can only be achieved by using high resolution mass spectrometry. The labor-intensive sample preparation activities have led to the application of automated procedures directed toward increasing sample throughput and optimizing use of the chemist’s time. Such sample preparation requires a specialized approach to quality control, including the strategic placement of system blanks. Such low levels of quantitation require care in controlling artifacts and laboratory contamination. The reporting of quantitative results corrected for percent lipid in the samples permits the correlation of quantitative results between different sample matrices. The laboratory must be carefully organized to insure sample coordination, monitoring of quality control, and timely reporting of results. Finally, sample collection in the field has been challenging, particularly in the case of adipose tissue.

Interest in toxic chemical exposure will continue to challenge the analytical chemist to provide better laboratory measurements for assessing exposure. We are developing a method for quantifying a number of volatile organic compounds in human whole blood. Concerns about exposure to this class of compounds has led to controls and monitoring programs on drinking water systems, a major source of human exposure. Our method development has defined the analytical approach as a variation on the traditional purge and trap technique. Work includes adapting instrumental hardware specifically for this sample matrix. Major challenges include a short-lived quality control material, quality control for a significant number of “nondetects” and electronic data handling for the large number of samples involved. The overall objective of this work is to estimate the presence of a number of important volatile compounds in whole blood for a specific population of 1200 people. The results of this study should make it possible to estimate the types and magnitude of exposure encountered by the U.S. population.

